# The Underlying Mechanism of Duanteng Yimu Decoction in Inhibiting Synovial Hyperplasia in Rheumatoid Arthritis

**DOI:** 10.1155/2023/2340538

**Published:** 2023-05-21

**Authors:** Wei Feng, Xiao-Qin Zhong, Xue-Xia Zheng, Qing-Ping Liu, Min-Ying Liu, Xiao-Bao Liu, Chang-Song Lin, Qiang Xu

**Affiliations:** ^1^The First Clinical Medicine School, Guangzhou University of Chinese Medicine, Guangzhou 510405, China; ^2^Department of Rheumatology, The First Affiliated Hospital of Guangzhou University of Chinese Medicine, Guangzhou 510405, China

## Abstract

Dysregulation of microRNAs (miRNAs) is associated with the pathogenesis of rheumatoid arthritis (RA). Our previous studies confirmed that Duanteng Yimu decoction (DTYMT) effectively inhibits RA fibroblast-like synoviocyte (FLS) proliferation. In this study, we investigated the influence of DTYMT on miR-221 in RA individuals. Hematoxylin–eosin (HE) staining was performed to assess histopathological alterations in collagen-induced arthritis (CIA) mice. The expression of miR-221-3p and TLR4 in PBMC, FLS, and cartilage was measured by RT-qPCR. In the in vitro experiments, DTYMT-containing serum was incubated with FLS-transfected miR-221 mimic or inhibitor. CCK-8 was performed to determine FLS proliferation, and the secretion of IL-1*β*, IL-6, IL-18, and TNF-*α* was quantified by ELISA assay. In addition, the regulation of miR-221 expression on FLS apoptosis was assessed using flow cytometry. Finally, western blot was employed to reflect TLR4/MyD88 protein levels. HE results showed that DTYMT effectively reduced synovial hyperplasia in the joints of CIA mice. RT-qPCR assay of FLS and cartilage of the model group showed that miR-221-3p and TLR4 significantly increased compared with those in the normal group. All outcomes were improved by DTYMT. The miR-221 mimic reversed the inhibitory effect of DTYMT-containing serum on FLS proliferation, the release of IL-1*β*, IL-18, IL-6, and TNF-*α*, and FLS apoptosis, as well as TLR4/MyD88 protein levels. The results showed that miR-221 promotes the activity of RA-FLS by activating TLR4/MyD88 signaling, and DTYMT treats RA by reducing miR-221 in CIA mice.

## 1. Introduction

Rheumatoid arthritis (RA) is a prevalent chronic and systemic inflammatory disorder with a global prevalence of approximately 0.5%–1.0% and is a higher incidence among women who are two to three times more likely to be affected than men [1, 2]. The primary features of RA are the expansion of synoviocytes, production of various cytokines, destruction of cartilage, and joint loss of physical ability [3, 4]. As RA progresses, the excessive accumulation of fibroblast-like synoviocytes in pathological joints ultimately leads to the production of cytokines responsible for bone and cartilage damage [5]. In addition, extra-articular manifestations and comorbidities were commonly seen in RA patients, including cardiovascular disease, pulmonary manifestations, gastrointestinal disorders, and neurologic involvement [6]. Patients with RA suffer from severe pain, fatigue, low quality of life, and higher mortality than osteoarthritis or normal people [7, 8].

MicroRNAs (miRNAs) are a class of noncoding RNAs, typically 21–24 nucleotides in length, which exert a vital function in regulating cellular processes such as differentiation, proliferation, and survival by targeting mRNA cleavage or translational inhibition [9, 10]. Several researches have documented the involvement of miR-221 in the inflammatory response [11, 12]. Aberrant miR-221 expression has been identified in patients with RA and has been considered to be relevant to disease activity [2, 13, 14]. Additionally, Quero et al. [11] described that increasing miR-221 in RA resulted in the conversion of M2-macrophages into M1 phenotype through inhibiting JAK3/STAT3 pathway activation [11]. The fibroblast-like synoviocyte (FLS) cell migration and invasion were inhibited if the miR-221 expression was downregulated [15].

Toll-like receptor 4 (TLR4) is a pivotal player in several immune-related pathologies [16]. Several researchers have described that TLR4 is highly expressed in chondrocytes and synoviocytes and contributes to the pathological process of skeletal muscle inflammatory diseases [17, 18]. In addition, TLR4 is closely related to changes in bone metabolism in rheumatoid arthritis, osteoarthritis, and other diseases [19–21]. MyD88 is a conductor of intracellular signal transduction, which could medicate the activity of TLR4 and ultimately promotes the secretion of proinflammatory factors [22, 23]. Thus, the TLR4/MyD88 signaling is crucial for innate immune regulation.

Duanteng Yimu decoction (DTYMD) is a Chinese medicine prescription used by our research group to treat RA. The formulation of DTYMD was *Tripterygium hypoglaucum* (H. Lév.) Hutch (30 g), *Leonurus japonicus* Houtt. (Lamiaceae) (30 g), and *Dipsacus asperoides* C. Y. Cheng et T. M. Ai (15 g). *Tripterygium* is considered one of the most effective traditional Chinese medicines (TCM) for treating RA due to its remarkable performance in immunosuppression, anti-inflammation, and cartilage protection [24]. Asperosaponin VI, extracted from *D. asperoides*, has been demonstrated to boost osteoblast differentiation, reduce the production of proinflammatory cytokines induced by IL-*β* in chondrocytes, and improve bone loss and cartilage inflammation [25, 26]. Additionally, Jung et al. [27] confirmed that its extract significantly alleviated joint inflammation in CIA mice. Leonurine, a main component of *D. asperoides*, has been proven to have multiple bioactivities, including antioxidant and anti-inflammatory effects [28]. It could inhibit the activation of NF-*κ*B induced by IL-*β* in osteoarthritis and subsequently the production of inflammatory factors, providing protective effects on cartilage and alleviating synovitis [29]. Our previous studies have revealed that DTYMD alleviates synovial inflammation and joint damage in RA models and reduces the migration and infiltration of fibroblasts in RA patients [30, 31].

Based on these findings, we hypothesized that DTYMD inhibits cartilage destruction in RA by modulating the miR-221-mediated TLR4 signaling pathway.

## 2. Materials and Methods

### 2.1. Experimental Animals and Model Construction

#### 2.1.1. Experimental Animals

Twenty-four 90-day-old male DBA/1 mice were obtained from the Southern Medical University and randomly separated into four groups (six mice per group): normal, collagen-induced arthritis (CIA) model, methotrexate (MTX), and DTYMT groups. The animals were housed under an SPF-grade condition with an alternating 12 hr light/dark cycle (temperature, 25 ± 4°C; humidity, 50%– 70%) and were given standard diet ad libitum.

#### 2.1.2. Animal Experimental Design

On day 0, the CIA model was guided by a previous study [32], animals, except the normal group, were first treated with 200 *μ*g of bovine type II collagen (cat. no. 20022, Chondrex Inc., Washington, USA) and complete Freudian adjuvant (cat. no. 7009, Chondrex Inc.) via a subcutaneous injection. On day 21, the mice were given a booster immunization with incomplete Freudian adjuvant (cat. no. 7002, Chondrex Inc.) and type II collagen. The next day, the mice were orally administered with MTX (1 mg/kg every 3 days), DTYMT (12.5 g/kg daily), or an identical volume of saline by gavage for 14 days. After the last day of administration, the blood samples were collected, then mice were euthanized via intrathecally injecting sodium pentobarbital (80 mg/kg). Their ankle joints were separated and placed in a 4% paraformaldehyde solution (cat. no. BL539A, Biosharp, Anhui, China). All animal experiments were approved by the Ethics Committee of the First Affiliated Hospital of Guangzhou University of Traditional Chinese Medicine (approval no. 2020006) and conducted in accordance with the guidelines established by the National Institutes of Health.

### 2.2. Hematoxylin-Eosin (HE) Staining

The ankle joints were immobilized in 4% paraformaldehyde, decalcified in 10% EDTA for approximately 5 weeks, embedded in paraffin, sliced into 4 *μ*m-thick slices, and stained with hematoxylin–eosin (HE) staining solution (cat. no. G1120, Solarbio, Beijing, China) following the manufacturer's description. After that, the joint slices were viewed under a light microscope (Olympus, Center Valley, PA, USA).

### 2.3. Cell Culture and Transfection

#### 2.3.1. Fibroblast-Like Synoviocytes (FLS): Isolation and Culture

According to the previous study [33], the synovial tissue of mice was cut into approximately 1 mm × 1 mm × 1 mm tissue blocks and then digested with 4 mg/ml type I collagenase at 37°C for 4 hr. Subsequently, the samples were centrifuged for 10 min (3,000 × *g*, 4°C), and the precipitate was mixed with Dulbecco's modified Eagle's medium (DMEM) contained with 10% FBS and cultured in a culture flask for 12 hr. The medium was changed on the second day before trypsin-EDTA digestion for 1 hr. Immediately after adding DMEM and the suspensions were centrifuged for 5 min (1,000 × *g*, 15°C) followed by resuspending using DMEM and cultured at 37°C with CO_2_.

#### 2.3.2. Cell Migration and Invasion Assays

Cell migration and invasion assays were performed in a 24-well transwell chamber (BD Biosciences, San Jose, CA, USA). Cells (1 × 10^5^) pretreated with DTYMT-containing serum (600 *μ*g/ml), IL-6 (30 ng/ml), or DTYMT-containing serum with IL-6 for 24 hr, were harvested and added to the upper chamber coated without or with Matrigel (BD Biosciences, San Jose, CA, USA). The upper and lower chambers were filled with serum-free medium and medium containing 10% FBS, respectively. After incubating at 37°C and 5% CO_2_ for 48 hr, the cells were fixed with methanol for 30 min after migration and invasion into the lower chamber insert and then stained with 0.1% crystal violet for 15 min. Cell counts were measured under an optical microscope (Olympus, Tokyo, Japan).

#### 2.3.3. Translation

Cells (2 × 10^5^) were added to a sixwell plate and incubated overnight (37°C, 5% CO_2_). Transfection was performed the following day with 100 nM of miR-221 mimics or inhibitors, including negative controls (NC), which were synthesized by Sangon Biotech Co., Ltd. Lipofectamine® 2000 (cat. no. 11668019, Invitrogen; Thermo Fisher Scientific, Inc.) was utilized for RNA transfection following the manufacturer's guidelines.

### 2.4. RT-qPCR Assay

Total RNA was extracted using TRIzol™ reagent (cat. no. 15596026, Thermo Fisher Scientific, Inc.) following the manufacturer's description, and cDNA was reverse transcribed using the PrimeScript RT reagent kit (code. no. RR047A Takara, Dalian, China). RT-qPCR analysis was conducted using SYBR® Premix Ex Taq™ II kit (cat. no. # RR820A, Takara Bio, Japan) and Applied Biosystems® 7500 Real-Time PCR System (CXF96, Bio-Rad, USA), using the following conditions: 50°C for 2 min, 95°C for 2 min, followed by 40 cycles of 95°C for 15 s and 60°C for 32 s. The primers used for RT-qPCR analysis are listed in Table 1. The 2^−*ΔΔ*Cq^ method was employed to quantify the relative mRNA expression.

### 2.5. Western Blot Assay

After the cells were lysed in lysis buffer (cat. no. R0030, Solarbio), the protein concentrations of these samples were measured using the BCA protein assay kit (cat. no. PC0020, Solarbio). Cellular proteins (20 *µ*g) were separated by SDS–PAGE gel (10%) and transferred onto PVDF membranes (cat. no. GVWP02500, Millipore Sigma). The membranes were rinsed with 10% TBS-Tween-20 (cat. no. T8220, Solarbio), blocked with 5% bovine serum albumin (cat. no. A8010, Solarbio), and were probed overnight at 4°C with primary antibodies. Subsequently, the blots were labeled with a secondary antibody for 2 hr at room temperature before visualization. The antibodies used are as follows: anti-TLR4 (1 : 1,000; cat. no. Ab13556; Abcam), anti-MyD88 (1 : 1,000; cat. no. ab219413; Abcam), secondary antibody (goat anti-rabbit; 1 : 10,000; cat. no. ab205718; Abcam), and anti-GAPDH (1 : 10,000; cat. no. ab181602; Abcam).

### 2.6. Cell Proliferation Assay

FLS and chondrocyte cells were added to 96-well plates (2.5 × 10^3^ cells/well) and cultured for up to 72 hr. The enzyme-labeled instrument (Thermo Fisher Scientific, Inc.) was utilized to determine the optical density values at 24 hr intervals using the Cell Counting Kit-8 (CCK8; cat. no. CK04, Solarbio) according to the manufacturer's recommendation.

### 2.7. Flow Cytometry Assay

In brief, Annexin V-FITC (5 *μ*l; cat. no. 556420, BD Biosciences) and propidium iodide (PI, cat. no. 556463) (10 *μ*l; BD Biosciences) were incubated with FLS and chondrocyte cells (5 × 10^5^ cells/ml) in the dark (15 min, 23 ± 2°C). Subsequently, the cells were washed twice with PBS (Gibco; Thermo Fisher Scientific, Inc.) and assessed by flow cytometry (BD Biosciences, San Jose, CA, USA).

### 2.8. Cytokine Quantification

In each experiment, the supernatant or serum from each subgroup of cells was collected to measure the levels of circulating IL-6, IL-1*β*, IL-18, and TNF-*α*. The evaluation was performed using commercial kits from R&D Systems (Minneapolis, USA), which included the mouse IL-1*β* ELISA kit, mouse IL-18 ELISA kit, mouse IL-6 Quantikine ELISA kit, and mouse TNF-*α* Quantikine ELISA kit.

### 2.9. Statistical Analysis

SPSS version 23.0 software (IBM SPSS, Armonk, NY, USA) was employed to perform all statistical analyses and the mean ± standard deviation was utilized to represent the data. The one-way analysis of variance (ANOVA) with a Bonferroni post hoc test and Student's *t*-test were employed to compare the differences between groups. Significance was set at *p* < 0.05. All experiments were conducted in triplicate.

## 3. Results

### 3.1. Effects of DTYMT on CIA Mice Joints

**Figure 1:**
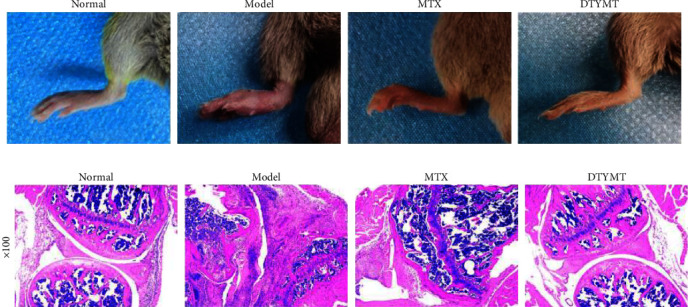
Effects of DTYMT on CIA mice. (a) The pictures of joint swelling in each group of mice after the last day of administration. (b) HE staining of ankle joints (magnification, ×100).

The collagen-induced arthritis is a typical RA model [34]. As shown in Figure 1(a), swollen joints were observed in the model group, indicating that our modeling was successful and could be used for subsequent experiments. Furthermore, after DTYMT treatment, the swollen joints of the model mice significantly improved, and the effects were comparable to those of MTX. These results suggested that DTYMT is effective against RA.

### 3.2. HE Staining

Compared with the normal group, the results showed severe damage to the cartilage surface, synovial hyperplasia, and inflammatory cell infiltration in mice of the model group (Figure 1(b)). However, these features were significantly ameliorated by DTYMT and MTX, suggesting that DTYMT inhibits synovial hyperplasia and the inflammatory response in CIA mice.

### 3.3. RT-qPCR Assay Results for miR-221 and TLR4 in PBMC

**Figure 2:**
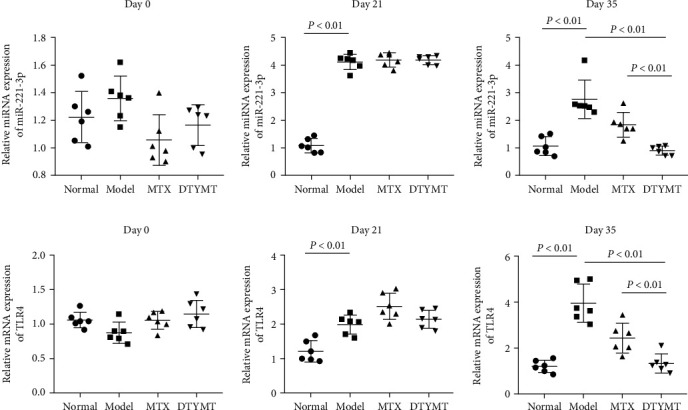
(a) RT-qPCR assay for miR-221-3p, and (b) TLR4 in PBMC of CIA mice. The first immunization was recorded as day 0, the second immunization was recorded as day 21, and the last day of administration was recorded as day 35. *n* = 6 per group.

As mentioned above, we were interested in miRNA changes in CIA mice. In this study, the mRNA level of miR-221-3p in PBMC was increased in CIA mice (Figure 2(a)). Furthermore, the expression of TLR4 in the model group was significantly elevated in relation to the normal group (Figure 2(b)). Treatment with DTYMT resulted in a significant reduction of miR-221-3p and TLR4 expression, and its efficacy was superior to that of MTX.

### 3.4. RT-qPCR Assay Results for FLS and Cartilage

**Figure 3:**
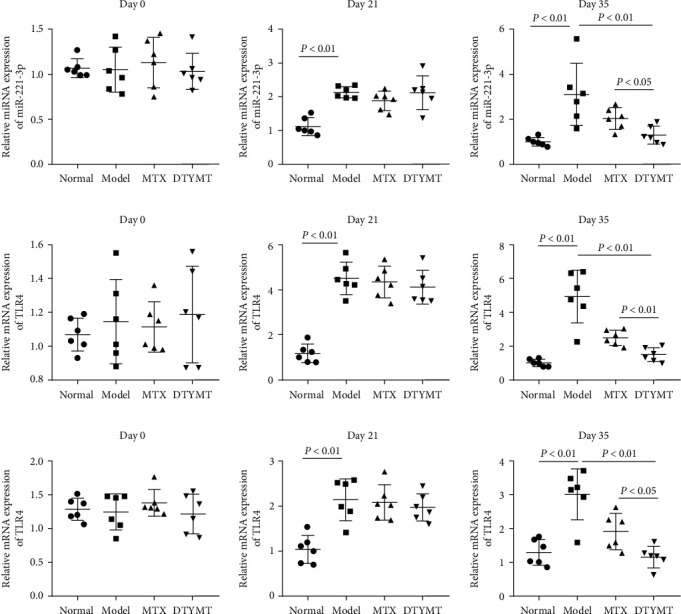
RT-qPCR assay for miR-221-3p and TLR4. (a) The expression of miR-221-3p in FLS. (b, c) The expression of TLR4 in FLS and cartilage. The first immunization was recorded as day 0, the second immunization was recorded as day 21, and the last day of administration was recorded as day 35. *n* = 6 per group.

One of the main characteristics of RA is the abnormal proliferation of FLS in the joint, which leads to cartilage damage. As expected, the mRNA levels of miR-221-3p and TLR4 were abnormally elevated in the FLS of CIA mice relative to normal mice (Figures 3(a) and 3(b)). Additionally, TLR4 expression was obviously elevated in the cartilage of model group (Figure 3(c)). Treatment with DTYMT improved the aberrant expression of miR-221-3p and TLR4 in CIA mice, which was better than that of MTX. Based on these results, we speculated that the reduction in synovial hyperplasia by DTYMT is correlated closely to its regulation on miR-221-3p and TLR4.

### 3.5. DTYMT-Containing Serum Inhibited Migration and Invasion of FLS

**Figure 4:**
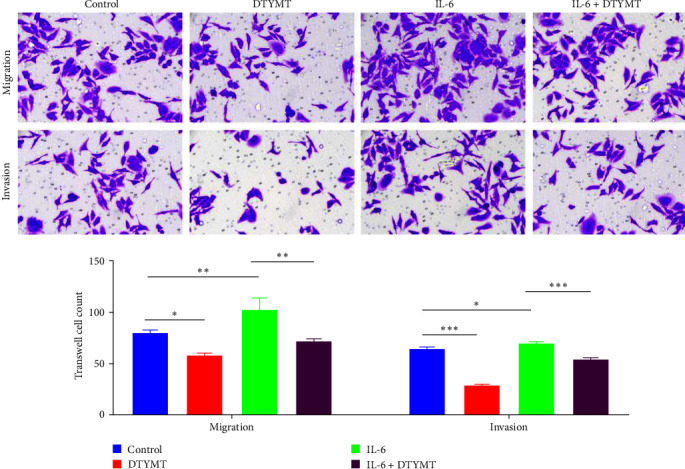
Transwell assays showed that DTYMT inhibited the migration and invasion of fibroblastic synovial cells and reversed the promotion of IL-6 on the cells (magnification, ×200).  ^*∗*^*p* < 0.05,  ^*∗∗*^*p* < 0.01,  ^*∗∗∗*^*p* < 0.001.

The FLS mobile capabilities were examined using a transwell assay to evaluate the effect of DTYMT on them. As shown in Figure 4, DTYMT-containing serum diminished the mobile capabilities of FLS. Additionally, DTYMT-containing serum partially counteracted the promoting effect of IL-6 on FLS.

### 3.6. Cytokine Quantification Results

**Figure 5:**
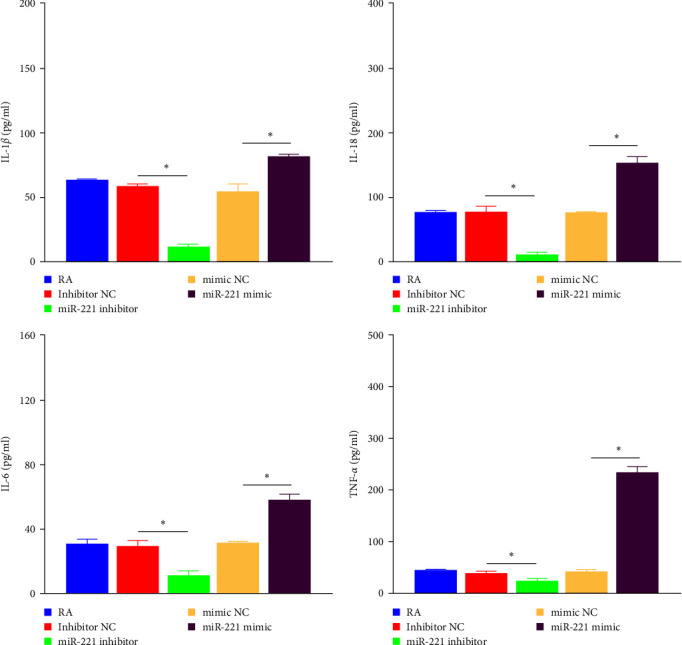
ELISA for the levels of IL-1*β*, IL-18, IL-6, and TNF-*α* in vitro experiments.  ^*∗*^*p* < 0.05.

The DTYMT-containing serum was incubated with RA-FLS transfected with the miR-221 mimic or inhibitor for 48 hr. The levels of IL-1*β*, IL-18, IL-6, and TNF-*α* in the supernatant were assessed by ELISA (Figure 5). Under DTYMT treatment, the miR-221 mimics rescued the production of these cytokines, which were reduced by DTYMT-containing serum. In addition, the miR-221 inhibitor significantly enhanced the inhibitory effects of DTYMT on these inflammatory factors.

### 3.7. CCK8 Results

**Figure 6:**
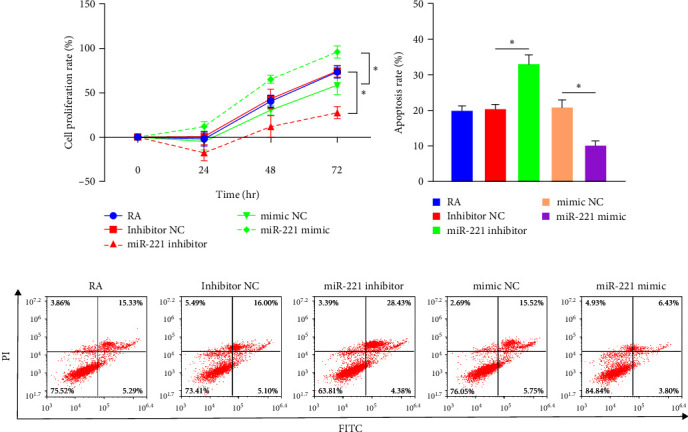
Regulation of miR-221 on the RA-FLS. (a) The proliferation of FLS by CCK8 analysis. (b) Flow cytometry assesses the apoptosis of FLS.  ^*∗*^*p* < 0.05.

CCK8 was used to measure the proliferation of FLS when miR-221 was overexpressed or inhibited. The outcomes showed that the inhibition of RA-FLS proliferation by DTYMT was weakened by miR-221 mimics, whereas the miR-221 inhibitor strengthened the inhibitory effects of DTYMT (Figure 6(a)).

### 3.8. Flow Cytometry Results

As shown in Figure 6(b), the miR-221 inhibitor increased FLS apoptosis, whereas the exact opposite results appeared in the miR-221 overexpression group. This evidence supports that under DTYMT treatment, the increase in miR-221 could promote the proliferation of FLS and reduce FLS apoptosis. Therefore, DTYMT attenuated FLS hyperplasia in CIA mice by inhibiting the expression of miR-221.

### 3.9. WB Results

**Figure 7:**
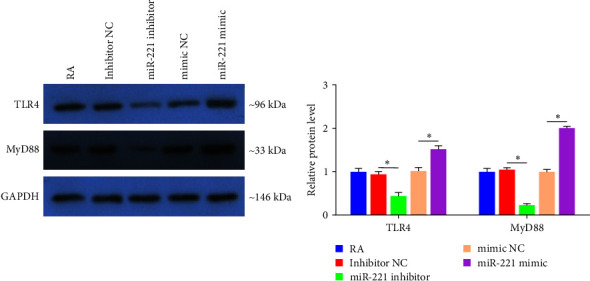
Regulation of miR-221 on TLR4/MyD88 signaling in FLS by western blot.  ^*∗*^*p* < 0.05.

We verified that miR-221 could regulate the function of RA-FLS; however, the underlying mechanism remains unknown. In vivo, TLR4 was observed to be closely related to miR-221; thus, we investigated the relationship between TLR4 and miR-221 using WB analysis. The miR-221 inhibitor transfection caused reduced expression of TLR4 and MyD88 in FLS, as demonstrated in Figure 7. However, the levels of TLR and MyD88 increased when RA-FLS were incubated with the miR-221 mimic. These results indicated that miR-221 mediates pathological activity by activating TLR4/MyD88 signaling.

## 4. Discussion

As the most common type of chronic arthritis, RA has received extensive attention worldwide. To date, nonsteroidal anti-inflammatory drugs (NSAIDs) alleviate pain in patients with RA only without stopping the deterioration of RA. Although disease-modifying antirheumatic drugs (DMARDs), such as methotrexate (MTX), leflunomide, sulfasalazine, and biologics, have great treatment potential and delay the progression of RA, long-term use is often accompanied by severe side effects or inadequate response, resulting in a heavy economic burden for RA patients [35, 36]. As a result, there is a pressing need for safer and more effective drugs to improve the management of RA.

Due to the cost-effective and multitarget effects, TCM has gained attention in the therapy of various diseases, including RA [37]. This study found that DTYMT effectively improved the CIA mice joints swelling and maintained the normal structure of joint tissues. FLS produces a lubricating hyaluronic acid-rich fluid in healthy joints and nourishes cartilage surfaces. However, in RA, abnormal proliferation of FLS transforms the inner layer of synovium into the pannus, mediates the production of MMPs, and damages the collagen-rich structures of joint tissues [38, 39]. In this study, we identified that miRNA-221 and TLR4 were highly increased in RA-FLS, PBMC, and cartilage but significantly reduced after DTYMT administration. These results suggest that miR-221 and TLR4 are involved in FLS-mediated lesions in RA. In addition, miR-221 is closely related to TLR4 expression. Zhang et al. [40] reported that miR-221 promoted TLR4 expression in *Mycoplasma pneumoniae*; however, another study [41] showed that increasing miR-221 inhibited TLR4 expression in human umbilical vein endothelial cells in atherosclerosis. At present, miR-221 appears mainly in a variety of cancer studies and has been suggested as a marker for the diagnosis or prognosis of cancer [42]. Nevertheless, there is still a poor understanding of miR-221 in treating RA. A report showed that downregulation of miR-221 in FLS reduced the production of proinflammatory cytokines and chemokines, inhibited cell mobile capability, and induced cell apoptosis by debilitating vascular endothelial growth factor (VEGF), matrix metalloproteinase-3 (MMP-3), and matrix metalloproteinase-9 (MMP-9) [15]. TLR4 exerts an essential effect in the development of RA by regulating the production of proinflammatory factors. Compared with healthy people or osteoarthritis (OA) patients, TLR4 is increased significantly [43], while the degradation of TLR4 has been found to alleviate symptoms of RA in mouse models [44].

To further investigate the relationship between miR-221 and TLR4 in chondrocyte injury mediated by RA-FLS, we modulated the expression of miR-221 in FLS by using a miR-221 inhibitor or mimic. As expected, the upregulation of miR-221 enhanced FLS proliferation, but a lower vitality of FLS was observed when inhibited mi-221. Persistent FLS accumulation in RA joints boosts the secretion of IL-1*β*, IL-6, TNF-*α*, and IL-18. IL-6 and TNF-*α* are regarded as central components in the synovial cytokine network of RA patients. These factors stimulate the formation and differentiation of osteoclasts, ultimately contributing to bone and cartilage degradation [45]. NOD-like receptor protein 3 (NLRP3) inflammasome is the most common inflammasome involved in the inflammation of RA. Activated NLRP3 inflammasome can result in the cleavage of pro-IL-1*β* and pro-IL-18, leading to the release of biologically active IL-1*β* and IL-18 [46]. Jing et al. [47] demonstrated that Celastrol, an extract from *T. wilfordii*, can inhibit the activation of NLRP3 inflammasome in RA, thereby alleviating inflammation symptoms. Similarly, IL-1*β* also participates in eliciting molecules associated with the generation of the inflammatory response as well as the activation of osteoclasts [48]. In addition, IL-18, like other proinflammatory cytokines, promotes the synthesis of nitric oxide, cell adhesion molecules, and chemokines, leading to the recruitment of more inflammatory cells to the joints [49]. In addition, IL-18 potentially accelerates bone damage by encouraging osteoclasts generation through the induction of TNF*α*, IL-1*β*, and IL-6 [50]. We verified that these cytokines were positively correlated with miRNA-221-transfected FLS. Inhibition of miR-221 in FLS contributes to the alleviation of inflammatory events. In contrast, upregulation of miR-221 in FLS led to the release of inflammatory factors. Furthermore, flow cytometry analysis demonstrated that miR-221 positively regulated FLS apoptosis.

We explored the potential mechanism by which DTYMT regulates miR-221-mediated FLS erosion of cartilage using WB analysis. As an anchoring adaptor protein, MyD88 processes and delivers the signals generated by the TLR and IL-1 receptor (TLR/IL-1R) superfamily [22] and is crucial in innate immune disease [51]. Park et al. [52] verified that inhibition of TLR4/MyD88 was associated with decreased recruitment of macrophages in CIA mice. Our data demonstrated that inhibition of miR-221 reduced the levels of TLR4 and MyD88 protein, whereas overexpression of miR-221 caused a higher expression of TLR4 and MyD88 protein, which suggests that DTYMT may mediate FLS attack on cartilage via the TLR4/MyD88 signaling pathway.

## 5. Conclusion

Taken together, we confirmed that DTYMT significantly improved the abnormal expression of miR-221and TLR4 and effectively alleviated joint inflammation in CIA mice. In vitro, miR-221 downregulation reduced FLS proliferation, alleviated inflammatory reactions, and promoted FLS apoptosis. This study indicated that DTYMT may mediate the activation of the TLR4/MyD88 pathway by regulating the miR-221 expression, which may have implications for the treatment of RA.

## Figures and Tables

**Table 1:** Summary of gene-specific real-time PCR primer sequences.

Gene		5′ to 3′ sequence	Size (bp)
TLR4	F	CCCAATTGACTCCATTCAAGC	229
	R	CCTGAACTCATCAATGCTCACAT	

miR-221-3p	F	ACACTCCAGCTGGGAGCTACATTGTCTGCTG	73

URP	R	CTCAACTGGTGTCGTGGA	

U6	F	CTCGCTTCGGCAGCACA	94
	R	AACGCTTCACGAATTTGCGT	

*β*-Actin	F	AGGGAAATCGTGCGTGACAT	150
	R	GAACCGCTCATTGCCGATAG	

## Data Availability

The datasets used and analyzed during the current study are available from the corresponding author upon reasonable request.
